# Deep and Shallow Water Effects on Developing Preschoolers’ Aquatic Skills

**DOI:** 10.2478/v10078-012-0037-1

**Published:** 2012-05-30

**Authors:** Aldo M. Costa, Daniel A. Marinho, Helena Rocha, António J. Silva, Tiago M. Barbosa, Sandra S. Ferreira, Marta Martins

**Affiliations:** 1 Sports Science Department of University of Beira Interior, Covilhã, Portugal.; 2 University of Trás-os-Montes and Alto Douro/CIDESD, Vila Real, Portugal.; 3 Polytechnic Institute of Bragança/CIDESD, Bragança, Portugal.; 4 Sports Science School of Rio Maior, Rio Maior, Portugal.; 5 Research Centre in Sports, Health and Human Development (CIDESD), Vila Real, Portugal.; 6 Department of Mathematics, University of Beira Interior, Covilhã, Portugal.; 7 Mathematics Center, University of Beira Interior, Covilhã, Portugal.

**Keywords:** aquatic skills, shallow water, deep water, teaching methods, preschoolers

## Abstract

The aim of the study was to assess deep and shallow water teaching methods in swimming lessons for preschool children and identify variations in the basic aquatic skills acquired. The study sample included 32 swimming instructors (16 from deep water programs and 16 from shallow water programs) and 98 preschool children (50 from deep water swimming pool and 48 from shallow water swimming pool). The children were also studied regarding their previous experience in swimming (6, 12 and 18 months or practice). Chi-Square test and Fisher’s exact test were used to compare the teaching methodology. A discriminant analysis was conducted with Λ wilk’s method to predict under what conditions students are better or worse (aquatic competence).

Results suggest that regardless of the non-significant variations found in teaching methods, the water depth can affect aquatic skill acquisition - shallow water lessons seem to impose greater water competence particularly after 6 months of practice. The discriminant function revealed a significant association between groups and all predictors for 6 months of swimming practice (p<0.001). Body position in gliding and leg displacements were the main predictors. For 12 and 18 months of practice, the discriminant function do not revealed any significant association between groups. As a conclusion, it seems that the teaching methodology of aquatic readiness based on deep and shallow water programs for preschoolers is not significantly different. However, shallow water lessons could be preferable for the development of basic aquatic skills.

## Introduction

Originally, infant swimming was undertaken to prevent possible drowning risks. On this, children under 5 years old have the highest drowning mortality rate for both genders (Peden & McGee, 2003). However, there is no scientific evidence that aquatic activities can diminish drowning risk (Asher *et al.,* 1995). Nevertheless, several studies (Bem *et al*., 2003; Zhao *et al.*, 2005; Courage *et al.,* 2006) observed clearly positive effects of aquatic programs in motor development.

Aquatic readiness includes fundamental skills, attitudes, and understandings that precede the acquisition of more advance aquatic skills, such as swimming strokes and water safety (Langendorfer & Bruya, 1995). This concept is particularly important because swimming is developed in a particular environment leading to spatial, temporal and energetic specific constrains (Marinho *et al.*, 2009). Thus, the starting point of developing aquatic readiness corresponds to the total inaptness to the aquatic environment and the lack of ability to perform intended propelling actions.

The traditional approach to teach swimming is sequential, following a fixed set of skills, standardized teaching progression, and leading up to a post test of new abilities (Erbaugh, 1986). Nevertheless, other approaches are known to be more synthetic, seeking to provide a compromise between the pleasure and technique through creative teaching procedures (such as water games) (Langendorfer *et al.,* 1988; Langendorfer & Bruya, 1995). Above all, it is intended that the acquisition of new movement patterns and aquatic behaviours, also provide an adequate stimulus to cognitive, affective and psychomotor development of children (Langendorfer, 1987; Martins *et al.*, 2010). This is particularly important while the optimal aquatic readiness period was identified to be between 5 and 6 years of age (Blanksby *et al.,* 1995; Pelayo *et al.,* 1997).

There are several factors that directly influence the organization of teaching and, therefore, determine their effectiveness (Zuo, 2004). According to some authors the main factors are (Murray, 1980; Langendorfer, 2010): (i) the number of children in the class, which usually does not exceed 10 children at an early stage; (ii) the didactic material, which allows a variation of stimuli; (iii) water temperature, which should range between 30º to 32º C; (iv) the number of classes per week, usually 2 times in ages ranging between 3 to 6 years old and; (v) the water depth, allowing a wide variety of aquatic experiences.

The water depth seems to be the only topic that adds some lack of consensus in the scientific and technical community. Indeed, to the best of our knowledge, few authors refer to the water depth as a determinant factor in teaching effectiveness (Murray, 1980). In a global concept approach, children had the chance to learn to swim at a swimming pool with two depths: the shallow for the initiation process (usually from 0.65 to 1.00 meter deep) and the deep for the advanced (usually from 1.00 meter to 2.00 meters deep). This method was characterized mainly by its analytic/progressive way to obtain the global gesture, where the basic aquatic skills were thought simultaneously, such as breathing, floating and displacement. However, on overall, the adaptation to the aquatic environment is held in shallow water swimming pools.

This study focuses on the process of aquatic skills achievement in these two different contexts - deep and shallow water swimming pools. So, the aims of the present study were two-fold: (i) to analyse the differences between teaching methods in deep and shallow water swimming lessons for 4–5 years old children; (ii) to determine deep and shallow water differences on developing preschooler’s aquatic skills, after 6, 12 and 18 months of practice.

## Material and Methods

### Participants

Data was collected during the month of June in four distinct swimming pools. There were 32 swimming instructors (29.3 ± 1.4 years old), responsible for the swimming lessons of the children included. All have appropriate academic qualifications for teaching swimming (i.e., a sports science graduation course) and were used as a convenience sample, according the depth of the swimming pool. Teachers were divided into two study groups, based on the type of facility used to conduct swimming lessons: sixteen held classes in deep water swimming pools (4.5 ± 1.2 years of professional experience) and the remaining 16 in shallow water swimming pools (5.4 ± 0.7 years of professional experience). Children were using in-water instruction and the ratio was eight children per class.

Ninety-eight children aged 4 years old (4.39 ± 0.49-yr) participated in this study. The children were also divided into two distinct groups, according to the type of facility they performed the swimming lessons as well (deep versus shallow water swimming pools). Fifty children participated in swimming lessons at deep water and 48 at shallow water. The sample was also studied according to previous experience of swimming: 6 months (16 subjects from deep water lessons and other 16 from shallow water lessons), 12 months (16 subjects from deep water lessons and 16 from shallow water lessons) and 18 months (16 subjects from deep water lessons and 18 shallow water lessons) of previous swimming lessons experience. Indeed, efforts were made to recruit subjects for making comparable groups. So, in advance, we ensure that all children had started swimming lessons at the same time (with previous 6, 12 or 18 months of experience) and had the same number of classes per week (2 times).

The progress was accessed retrospectively, by an observation table of the aquatic motor skills acquired, adapted from Langendorfer & Bruya (1995) and Langendorfer *et al.* (1987). Since boys and girls demonstrate fairly similar rates of motor and cognitive development during childhood they were combined in this research (Eckert, 1987). The participants’ parents and teachers provided written informed consent to participate in this study, and the procedures were approved by the institutional review board.

### Measures

A questionnaire was applied to the teachers in order to access the teaching methodology, based on Birmingham & Wilkinson (2003) recommendations for the construction of questionnaires. The final version of the questionnaire included the following items: (i) *What teaching purposes are important in aquatic readiness lessons* (agree or not agree answer) - survive in water; learning any formal strokes; no fear of the water; promote an enjoyable activity; develop future competitive swimmers; (ii) *How much the didactic material was used* (for each proposed material, the teacher’s answer is possible in four ways - always used; sometimes, rarely and never): none, flutter boards, swimming armbands, swimming noodle, water small sticks, rings or small hoops (not floating); (iii) *Which aquatic skills were more important in the planned lessons* (the answer was also possible in four ways - always developed; sometimes, rarely and never): water entry, water orientation and adjustment, submersion, buoyancy, leg patterns, combined movements, glides, twists and turns, directed splashing and breath control.

All children included in the study sample were evaluated for their aquatic readiness, using an original observation form field of aquatic motor skills, based on Langerdorfer *et al.* (1987) Hence, the aquatic motor skills assessed were the following: water entry (*Sk1*); water orientation and adjustment at vertical position (*Sk2*); breath control - immersion of the face and eye opening (*Sk3*); horizontal buoyancy (*Sk4*); body position at ventral gliding (*Sk5*); body position at dorsal gliding (*Sk6*); body position at longitudinal rotation in gliding (*Sk7*); body position at front and back somersaults (*Sk8*); leg kick with breath control at ventral body position, with flutter boards (*Sk9*) and without any flutter device (*Sk10*); (ix) leg kick with breath control at dorsal body position with flutter boards (*Sk11*) and without any flutter device (*Sk12*); feet-first entry (*Sk13*); head-first entry (*Sk14*); (x) Autonomous in deep pool (legs and arms displacement) (Sk15); vertical buoyancy at deep water (*Sk16*) and; deep-water immersion (*Sk17*). Each of these skills was divided into increasing levels of complexity, as proposed by Langendorfer & Bruya (1995). As such, each of the 17 skill categories described above includes three, four or even five levels of complexity, depending on the category – enable to perform at stage one, rudimentary movements at stage two (or three) and fundamental movements at stage three (or even four or five) that precede the specific motor skills acquisition. The children studied had three attempts to achieve the proposed exercises.

### Procedures

The questionnaire was tested prior its application. Subsequently, minor adjustments were made to increase the clarity of the questions included. The questionnaire was still subject to detailed review by 3 experts in swimming pedagogy. Then, the same investigator administered the questionnaire to each swimming instructor.

The observation form field of aquatic motor skills was firstly tested in 6 children not covered in this study. Later, a swimming instructor with academic qualifications, who was unaware of the present study, applied the same observation form to the same subjects. The inter-rater agreement was calculated.

All sessions had 40 minutes duration. Water temperature was 30 ºC, air temperature 29 ºC and 65% of humidity. To increase the objectivity of the assessment, the instruction for children to perform each aquatic skill was given by the same experienced researcher. For each student included in the sample, was filled in an observation form. All observation forms were completed by the same subject, from 17–20 pm. The information for the exercises was given by the same subject and always with the same feedback. Three attempts were given to perform the exercises proposed.

### Statistical analysis

Descriptive statistics adopted were the mean and standard deviation.

Cohen’s kappa coefficient, a statistical measure of inter-rater agreement was calculated for aquatic motor skills evaluations. The differences between groups regarding the teaching methodology were compared by Chi-Square test; the Fisher’s exact test was also used when appropriate. A discriminant analysis was conducted with Λ wilk’s method (Ferreira et al., 2011) to predict under what conditions (shallow-water or deep-water) a student will have better or worse aquatic readiness (all variables together method). Predictor variables were the aquatic motor skills previously described (*Sk1*, *Sk2*, *Sk3*,..., *Sk17*). Box’s M was used to test the assumption of homogeneity of covariance matrices. Statistical significance was set at p<0.05.

## Results

The questionnaire responses from both groups of instructors surveyed (table 1) in relationship to the purposes of their aquatic readiness lessons (table 1) were not different (*water survival,* p=1.0; *learning to swim,* p=0.11; *not fear the water*, p=0.33; *swimming for fun,* p=1.0 *and to obtain professional swimmers*, p=0.17).

However, deep-water instructors seem to overestimate the learning of a formal swimming technique and presented differences concerning the “fear of the water” as an activity goal (p<0.05).

Table 2 shows the teacher’s responses about the use of support material and the contents applied in shallow and deep water; showing the differences between monitors (intra group results). Because the expected frequency in some categories was less than one, it was not possible to proceed with inferential analysis. Even so, one can note that for each didactic material, the most common response to the level of use was “sometimes”; which reveals a great variety in the use of different didactic equipment by both groups of instructors. Regarding to the contents applied in both water programs, we observed a noticeable variability in the items: combined movements (56.6% of deep water instructors “always” develop this skill, whereas this prevalence is only visible in 18% of their shallow colleagues), glides (developed “always” by 81.3% of the shallow water instructors, while just 31.3% of deep water instructors include this skill constantly) and breath control (all shallow water instructors “always” develop this aquatic readiness skill, whereas only 56.3% of their deep-water colleagues usually add this skill in their swimming lessons). Besides these observed differences, one can note that “twists and turns” is the less valued aquatic skill by all swimming instructors surveyed.

The degree of agreement among aquatic readiness examiners (inter-rater agreement) was high (Kappa=0.90; p<0.001), showing that our observation form field could be appropriate for measuring aquatic readiness by trained raters.

Table 3 shows the aquatic skills acquired by shallow-water and deep-water students after 6, 12 and 18 months of practice. The data suggest that shallow water lessons seem to impose greater water competence particularly after 6 months of practice. Indeed, after 6 months of practice there are significant differences (p<0.01) between the means of both groups on most aquatic motor skills assessed. After 12 months of practice the level of achievement are still significant lower for deep-water students in several aquatic skills. After 18 months of swimming practice, students have a higher number of aquatic skills acquired (a higher level of complexity for each skill); however, no significant differences in aquatic readiness were found between deep and shallow water programs.

The discriminant function revealed a significant association between groups and all predictors only for the 6 months of swimming practice, accounting for (0.932)^2=87% between group variability (Λ=0.131, Qui^2=43.778, p<0.001). Moreover, closer analysis of the structure matrix (table 4.1) revealed some significant predictors (the first four variables ordered by absolute size of correlation within function): body position at ventral gliding (*Sk5*, r=0.467); body position at dorsal gliding (*Sk6,* r=0.441); leg kick with breath control at dorsal body position with flutter boards (*Sk11, r=0.417*) and without any flutter device (*Sk12, r=0.413*). The cross-validated classification showed that overall 81.3% were correctly classified.

In the other cases, for 12 and 18 months of practice, the discriminant function did not reveal any significant association between groups; for 12 months it was found a Λ=0.395, Qui^2=19.945 (p=0.277) and for 18 months a Λ=0.488, Qui^2=17.240 (p=0.370).

The table functions at groups centroids (table 4.2) is used to establish the *cutting point* for classifying cases. If the two groups are of equal size, the best cutting point is half way between the values of the functions at group centroids (that is, the average). If the groups are unequal, the optimal cutting point is the weighted average of the two values. For 6 months of practice the function does discriminate well (the classification results reveal that 100.0% of students were correctly classified); deep-water students were classified with slightly better accuracy (87.5%) than shallow-water (75.0%). Shallow-water students have a mean of 2.50 (±0.917) while deep-water students produce a mean of −2.50 (±1.076).

For 12 months of practice the function does discriminate well (the classification results reveal that 84.4% of students were classified correctly into shallow-water or deep-water); deep-water students were classified with a little better accuracy (50.0%) than their colleagues (43.8%).

The classification results for 18 months of practice reveal that 79.4% of students were classified correctly into shallow-water or deep-water. Here, shallow-water students were classified with slightly better accuracy (61.1%) than deep-water students (56.3%). For 18 months of practice, shallow-water students have a mean of 0.94 (±1.013) while deep-water students produce a mean of −1.05 (±0.986).

## Discussion

The first aim of the present study was to describe the differences in the teaching methodology of aquatic readiness between deep and shallow water programs for preschool children. Our results suggest no significant differences between both teaching methods. The second main objective was to determine aquatic readiness differences between deep and shallow water students after 6, 12 and 18 months of practice. In this respect, discriminant function analysis showed a significant association between both groups and all predictors (aquatic skills) only for 6 months of swimming practice. However, even up to 12 months of practice, shallow-water students seem to have a higher level of aquatic competence in quite a few relevant skills. This could mean that deep-water programs for preschoolers should be carefully planned in order to experience more intensely certain basic skills that are less stimulated due to the context of practice. Body position during gliding is one of these skills with very low success among deep-water students.

Analyzing the descriptive data regarding the teaching methodology (tables 1 and 2), one can note some important variations that should be emphasized. The first variation appears to occur in the teaching purposes of aquatic readiness programs, as deep-water swimming teachers seem to overvalue the learning of a swimming stroke (87.8%). Actually, without a flotation device or minimal adult assistance, the child presents difficulties to independently propel him/herself in deep-water swimming pools (Langendorfer & Bruya, 1995). So, the search of propulsive autonomy, even by dog paddle or rudimentary crawl, may prove to be an earlier goal in deep water lessons. However, the development changes in aquatic motor patterns, having as main intention the propulsive autonomy, cause a regressive swimming program, apart from the vast knowledge explosion occurring in fields from neurophysiology to pedagogy (Langendorfer & Bruya, 1995). Moreover, swim schools need also to counter parental misconceptions of the role of swimming lessons for preschool children in drowning prevention and reiterate the importance of close adult supervision of children around water (Moran & Stanley, 2006).

Thus, the fact that swimming armbands have a more frequent use by deep-water instructors is hardly surprising, especially if used in the early stages of their swimming program. The most common equipment classification distinguishes it based on the primary purpose for which is intended, i.e. on the skill that is deliberately facilitated. However, it should be noted that any material could reach more than one educational purpose. To our knowledge, the literature is limited on studies regarding the advantages (or disadvantages) of the use of didactic material (including flotation devices) in the teaching of swimming. In one of those scarce studies, Erbaugh (1986) found that horizontal body position is first reached when using auxiliary materials. Nonetheless, aquatic instructors are increasingly distant from its overuse. Indeed, the use of swimming armbands or float vests in the quest to developing buoyancy competence or even body position patterns is criticized by several other authors (Blanksby *et al.,* 1995, Langerdorfer, 1987).

Our results also show a noticeable variability in the importance given to certain aquatic skills between both groups of teachers, especially the skills *combined movements* (more important to deep water teachers), *glides* and *breath control* (more important to shallow water instructors). As mentioned above, since children cannot reach the bottom of the pool, it seems expected that the search of a fair combination of movements, particularly a combined propulsive action of legs or arms with breath control, played a major goal on this kind of classes. The opinion expressed by all swimming instructors in the current study that body rotations such *twists and turns* is the less valued aquatic skill seems inadequate to the opinion of several authors and/or swimming textbooks (Langendorfer & Bruya, 1995) for the full development of aquatic competence. Rotations are no more than momentary or sporadic changes of the body balance acquired in water, performed on different planes of motion (sagittal, frontal and transverse). Thus, the development of such basic aquatic skill during preschool period will be decisive for later and more advanced swimming skill and stroke acquisition (Erbaugh, 1978; Langendorfer & Bruya, 1995).

The second aim of this study was to identify variations in the basic aquatic skills acquired between deep and shallow water students, after 6, 12 and 18 months of practice. Yet, first, it is important to mention that children with more practice (i.e., 12 and 18 months) seem to have a higher number of water skills acquired, although we do not have any comparative statistical data on this matter. In fact that it would be an excellent proposal for future work in this area. Observing table 3, the shallow water students after 6 months of swimming lessons seem to have a greater aquatic competence in nearly all the aquatic skills measured (p<0.01). In fact, the discriminant function revealed that groups of students (shallow-water *versus* deep-water students) are significant unequal (p<0.001) but only at 6 months of practice. Indeed, table 4.1, shows a good discriminant function since most cases are near the mean and there is a clear separation on the two sides. In table 4.2, the structure matrix revealed some significant predictors with a relevant absolute size of correlation within function (*Sk5*, *Sk6*, *Sk11*, *Sk12*). After 12 and 18 months of practice the discriminant function revealed that both groups of students are of equal size (p=0.277, p=0.370, respectively). Nevertheless, it seems important to emphasize that up to 12 months of practice, deep-water students are still unable to perform a streamlined glide (*Sk6* and *Sk7*), to maintain good body position at horizontal buoyancy (*Sk4*) or even to perform leg kick with breath control at ventral or dorsal positions without any flutter device (*Sk10* and *Sk12*). Differences (deep water instructors and shallow water instructor) could be due to the diversity of methodologies, which can influence the acquisition of different evaluated skills (Langendorfer & Bruya, 1995) that can be considered a limitation of the research.

In our opinion, when possible, a combination between deep and shallow water swimming lessons seems to represent an important scope to this kind of aquatic program. The neurophysiologic readiness of the body in perform determined skills can be in the origin of related differences. In fact, in can be non-productive demanding 4 years old children to have an idiokinetic segmental control. Unfortunately, for the best of our knowledge, at the moment there is no study regarding this issue.

## Conclusion

In conclusion, our results suggest that the teaching methodology of aquatic readiness based on deep and shallow water programs for preschool children it is not significantly different. Propulsive autonomy seems to be overvalued by deep-water swimming instructors. For that reason, conceivably, buoyancy armbands have a more frequent use by these instructors. Despite the tenuous methodological bias, water depth seems to negatively affect aquatic skill acquisition in preschool children. Indeed, the discriminant function revealed a significant association between groups and all predictors for 6 months of swimming practice, analyzing all variables together. Body position at ventral and dorsal gliding and leg displacements at dorsal body position (with and without any flutter device) were the main significant predictors. Based on this study, one can evidence the importance of shallow water (alone and possibly in combination with deep-water experiences) for the development of some basic aquatic skills.

## Figures and Tables

**Table 1:** Teacher’s responses about the purpose of their aquatic readiness lessons

	**Shallow water instrutors (n=16)**		**Deep water instructors (n=16)**		**p-value**
	Agree	Not Agree	Agree	Not Agree	
Water survival	14 (87,50%)	2 (25,0%)	15 (93,75%)	1 (6,25%)	1.0
Learning to swim	9 (56,25%)	7 (43,75%)	14 (87,75%)	2(12,5%)	0.11
Not fear the water	12 (75,0%)	4 (25,0%)	15 (93,75%)	1 (6,25%)	0.33
Swimming for fun	16 (100,0%)	0 (0%)	16 (100,0%)	0 (0,0%)	1.0
To develop future swimmers	1 (6,25%)	15 (93,75%)	5 (31,25%)	11 (68,75%)	0.17

**Table 2:** Teacher’s responses about the use of support material and the contents applied in shallow and deep water

***Material***	**Shallow water instrutors (n=16)**				**Deep water instructors (n=16)**			
	*Always*	*Sometimes*	*Rarely*	*Never*	*Always*	*Sometimes*	*Rarely*	*Never*
None	1 (6,25%)	6 (37,5%)	5 (31,25%)	4 (25,0%)	0 (0,0%)	9 (56,25%)	5 (31,25%)	2 (12,50%)
Swimming Board	3 (18,75%)	12 (75,0%)	1 (6,25%)	0 (0,0%)	4 (25,0%)	12 (75,0%)	0 (0,0%)	0 (0,0%)
Arm band	0 (0,0%)	2 (12,5%)	4 (25,0%)	10 (62,25%)	1 (6,25%)	6 (37,5%)	2 (12,5%)	7 (43,75%)
Floating tube	3 (18,75%)	11 (68,5%)	0 (0,0%)	2 (12,5%)	4 (25,0%)	12 (75,0%)	0 (0,0%)	0 (0,0%)
Sinking toys	3 (18,75%)	11 (68,5%)	1 (6,25%)	1 (6,25%)	2 (12,5%)	13 (81,25%)	1 (6,25%)	0 (0,0%)
Others	3 (18,75%)	9(56,25%)	3 (18,75%)	1(6,25%)	2 (12,5%)	12 (75,0%)	0 (0,0%)	2 (12,5%)

***Skills***								

Water entry	13 (81,25%)	3 (18,75%)	0 (0,0%)	0 (0,0%)	10 (62,5%)	6 (37,5%)	1 (6,25%)	0 (0,0%)
Confidence & Security	14 (87,5%)	2 (12,5%)	0 (0,0%)	0 (0,0%)	13 (81,25%)	2 (12,5%)	0 (0,0%)	1(6,25%)
Imersion	8 (50,0%)	3 (18,75%)	0 (0,0%)	0 (0,0%)	8 (50,0%)	8 (50,0%)	5 (31,25%)	0 (0,0%)
Balance	12 (75,0%)	4 (25,0%)	0 (0,0%)	0 (0,0%)	10 (62,5%)	6 (37,5%)	0 (0,0%)	0 (0,0%)
Legs displacement	10 (62,5%)	4 (25,0%)	2 (12,5%)	0 (0,0%)	14 (87,5%)	2 (12,5%)	0 (0,0%)	0 (0,0%)
Arms & legs displacement	3 (18,75%)	7 (43,75%)	6 (37,5%)	0 (0,0%)	9 (56,25%)	5 (31,25%)	2 (12,5%)	0 (0,0%)
Glide	13 (81,25%)	2 (12,5%)	1 (6,25%)	0 (0,0%)	5 (31,25%)	11 (68,5%)	0 (0,0%)	0 (0,0%)
Rotations	3 (18,75%)	11 (68,5%)	2 (12,5%)	0 (0,0%)	2 (12,5%)	12 (75,0%)	2 (12,5%)	0 (0,0%)
Basics	7 (43,75%)	6 (37,5%)	2 (12,5%)	1 (6,25%)	8 (50,0%)	8 (50,0%)	0 (0,0%)	0 (0,0%)
Dives	11 (68,5%)	4 (25,0%)	2 (12,5%)	0 (0,0%)	9 (56,25%)	7 (43,75%)	0 (0,0%)	0 (0,0%)
Breath control	16 (100,0%)	0 (0,0%)	0 (0,0%)	0 (0,0%)	9 (56,25%)	7 (43,5%)	0 (0,0%)	0 (0,0%)
Depth imersions	0 (0,0%)	10 (62,5%)	1 (6,25%)	1 (6,25%)	9 (56,25%)	7 (43,75%)	0 (0,0%)	0 (0,0%)

**Table 3:** Aquatic skills acquired (Mean ± SD) by shallow-water and deep-water students after 6, 12 and 18 months of practice

	**6 months of practice**				**12 months of practice**			**18 months of practice**		

**Skill**	**Levels of complexity**	**Shallow-water**	**Deep-water**	p-value	**Shallow-water**	**Deep-water**	p-value	**Shallow-water**	**Deep-water**	p
		
		Mean ± SD	Mean ± SD		Mean ± SD	Mean ± SD		Mean ± SD	Mean ± SD	
*Sk1*	1 to 3	3,00±0,00	2,63±0,50	.005 ^*^	3,00±0,00	2,88±0,34	.154	3,00±0,00	3,00±0,00	
*Sk2*	1 to 3	2,88±0,34	2,63±0,50	.109	2,75±0,45	2,75±0,45	1.000	2,89±0,32	2,88±0,34	.904
*Sk3*	1 to 5	4,38±0,89	4,06±0,93	.338	4,06±1,06	4,31±0,95	.488	4,33±0,91	4,56±0,81	.446
*Sk4*	1 to 4	3,13±1,03	1,38±0,72	.000 ^*^	2,63±1,09	1,75±0,86	.017 ^*^	3,11±0,96	2,69±1,20	.261
*Sk5*	1 to 4	3,50±0,82	1,50±0,89	.000 ^*^	2,88±1,09	2,19±1,38	.128	3,39±0,85	2,81±1,17	.107
*Sk6*	1 to 4	2,88±1,20	1,00±0,00	.000 ^*^	2,69±1,25	1,63±1,03	.013 ^*^	2,83±1,25	2,13±1,26	.110
*Sk7*	1 to 3	1,94±0,57	1,06±0,25	.000 ^*^	1,94±0,68	1,31±0,48	.005 ^*^	2,00±0,59	1,81±0,75	.423
*Sk8*	1 to 4	1,94±1,24	1,06±0,25	.009 ^*^	1,44±0,81	1,13±0,50	.201	1,89±1,18	1,50±0,97	.305
*Sk9*	1 to 4	2,88±0,89	1,44±0,51	.000 ^*^	2,63±1,09	2,13±0,81	.150	2,94±0,87	2,81±0,91	.669
*Sk10*	1 to 4	2,63±0,81	1,31±0,48	.000 ^*^	2,56±0,96	1,81±0,75	.020 ^*^	2,67±0,84	2,31±0,87	.237
*Sk11*	1 to 4	2,38±0,62	1,25±0,45	.000 ^*^	2,44±1,21	2,06±0,93	.333	2,44±0,62	2,44±0,89	.979
*Sk12*	1 to 4	2,25±0,78	1,06±0,25	.000 ^*^	2,31±1,08	1,44±0,63	.009 ^*^	2,33±0,84	2,31±1,08	.950
*Sk13*	1 to 3	2,69±0,60	2,13±0,62	.014 ^*^	2,63±0,50	2,31±0,48	.081	2,72±0,58	2,56±0,51	.401
*Sk14*	1 to 3	2,44±0,51	1,69±0,79	.003 ^*^	2,44±0,51	2,19±0,75	.280	2,44±0,51	2,50±0,63	.779
*Sk15*	1 to 3	2,19±0,83	1,75±0,68	.115	1,63±0,81	2,19±0,54	.028 ^*^	2,28±0,83	2,63±0,62	.180
*Sk16*	1 to 5	3,06±1,48	1,44±0,96	.001 ^*^	2,75±1,48	2,88±1,86	.835	3,28±1,53	3,19±1,72	.872
*Sk17*	1 to 4	2,75±1,18	1,38±0,72	.000 ^*^	1,81±1,05	2,25±1,65	.378	2,89±1,18	2,56±1,32	.452

(*) p<0.05

**Table 4.1:** Structure matrix for shallow-water and deep-water students after 6, 12 and 18 months of practice

**6 months of practice**		**12 months of practice**		**18 months of practice**	

**Predictors**	**Function**	**Predictors**	**Function**	**Predictors**	**Function**
*Sk5*	.467	*Sk7*	.444	*Sk5*	.286
*Sk6*	.441	*Sk12*	.414	*Sk6*	.284
*Sk11*	.417	*Sk6*	.388	*Sk15*	−.237
*Sk12*	.413	*Sk4*	.373	*Sk10*	.208
*Sk9*	.398	*Sk10*	.363	*Sk4*	.197
*Sk10*	.396	*Sk15*	−.342	*Sk8*	.180
*Sk7*	.396	*Sk13*	.267	*Sk13*	.147
*Sk4*	.396	*Sk5*	.231	*Sk7*	.140
*Sk17*	.281	*Sk9*	.218	*Sk3*	−.133
*Sk16*	.260	*Sk1*	.216	*Sk17*	.131
*Sk14*	.225	*Sk8*	.193	*SK9*	.074
*Sk1*	.212	*Sk14*	.163	*Sk14*	−.049
*Sk8*	.196	*Sk11*	.145	*Sk16*	.028
*Sk13*	.184	*Sk17*	−.132	*Sk2*	.021
*Sk2*	.117	*Sk3*	−.104	*Sk12*	.011
*Sk15*	.115	*Sk16*	−.031	*Sk11*	.005
*Sk3*	.069	*Sk2*	.000		

Pooled within-groups correlations between discriminating variables and standardized canonical discriminant functions. Variables ordered by absolute size of correlation within function.

**Table 4.2:** Functions at Group Centroids for shallow-water and deep-water students after 6, 12 and 18 months of practice

	**6 months of practice**	**12 months of practice**	**18 months of practice**

**Context of practice**	**Function**	**Function**	**Function**
*Deep-water*	−2.499	−1.197	−1.055
Shallow-water	2.499	1.197	.938

Unstandardized canonical discriminant functions evaluated at group means.
